# Reaching global HIV/AIDS goals: What got us here, won't get us there

**DOI:** 10.1371/journal.pmed.1002421

**Published:** 2017-11-07

**Authors:** Wafaa M. El-Sadr, Katherine Harripersaud, Miriam Rabkin

**Affiliations:** ICAP at Columbia University, Mailman School of Public Health, New York, New York, United States

## Abstract

In a Perspective, Wafaa El-Sadr and colleagues discuss tailored approaches to treatment and prevention of HIV infection.

## Much accomplished, but much more needed

A decade ago, today’s progress towards confronting the global HIV epidemic would have been unimaginable. A remarkable global mobilization of resources through the United States President’s Emergency Plan for AIDS Relief (PEPFAR) and the Global Fund for AIDS, Tuberculosis and Malaria, combined with the commitment of affected countries and communities, has enabled 19.5 million persons living with HIV (PLHIV) to access life-saving antiretroviral therapy (ART) [1]. This has resulted in decreasing HIV-related morbidity and mortality and contributed to a significant decline in the number of new infections [1].

Despite earlier concerns about the feasibility of scaling up HIV services in resource-limited settings, the majority of PLHIV accessing ART are in sub-Saharan Africa, where many countries have austere health systems characterized by scarce healthcare providers and weak laboratory, infrastructure, drug procurement, monitoring, and governance systems [2]. One of the critical enablers of this achievement was the adoption of the public health approach to HIV service delivery [2]. This strategy used simple evidence-based algorithms for HIV testing, prevention, and treatment; employed a single first-line antiretroviral regimen, standardized laboratory tests, and testing schedules; and involved streamlined data monitoring systems [3]. The simplicity and consistency of this approach enabled HIV services to be provided by nonphysician clinicians and facilitated the establishment of simplified laboratory and medication procurement systems, enabling the successful scale-up of treatment [3].

By facilitating the successful scale-up of HIV services, the public health approach is arguably “what got us here,” to a context in which more than half of all PLHIV are accessing treatment [1]. However, in order to reach ambitious global targets and achieve epidemic control, much more must be done—and swiftly. Not only must the number of PLHIV accessing ART increase substantially to reach 30 million people by 2020, but the quality of HIV services must be enhanced and effective primary prevention interventions must be brought to scale [1]. Challenges include reaching diverse patient populations, retaining them in either treatment or prevention programs, supporting adherence to ART and prevention methods, and addressing long wait times and health facility crowding, a problem for both recipients of care and health workers. In addition, concern over the plateauing of global resources highlights the vital importance of efficiency and cost-effectiveness as a possible way to address this enormous challenge [1].

## Differentiated care for people living with HIV

Differentiated care may be an important step towards addressing health system and individual barriers to achieve HIV treatment goals [4]. Whereas earlier efforts, anchored in the public health approach, often distinguished only 2 groups of adult patients—pregnant and nonpregnant—differentiated care models tailor service frequency, service location, service intensity, and type of service provider for more categories of PLHIV [4]. The goal of differentiated care is to provide client-centered services that encourage engagement, adherence, and retention in care while also maximizing efficiency.

The most urgent need has been to develop models of care for PLHIV who are stable on ART, generally defined by high adherence, evidence of favorable immunological response, and/or virological suppression [5]. By their sheer numbers, such patients represent the vast majority of visits to health facilities and contribute the most to provider workload, despite the fact that they do not require frequent clinical assessment. Moreover, requiring stable patients to repeatedly return to health facilities overlooks their needs and priorities and may itself be a barrier to retention in care and adherence to treatment. Differentiated care for stable patients includes group models, such as facility-based adherence clubs and community-based antiretroviral groups, as well as individual models, such as facility-based fast-track appointments, increased visit spacing, and community-based ART pickup [6]. These approaches recognize that successful treatment of a chronic disease, such as HIV, depends on patient self-management, often enhanced by the support provided by families and communities [6].

There is also high interest in developing differentiated care for other groups of PLHIV including pregnant women, PLHIV with advanced HIV disease, adolescents, men, migrant and mobile populations, and key populations, such as men who have sex with men, sex workers, and people who inject drugs. Although these groups bear a disproportionate burden of HIV infections, many face structural and psychosocial barriers, such as stigma, discrimination, and insensitive providers, that stand in the way of achieving optimal access to and engagement with care [7]. Other groups of PLHIV face difficulties in remaining in care due to competing priorities. For example, while the scale-up of ART for pregnant HIV-positive women has been impressive, their retention in care, particularly postpartum, remains suboptimal [8]. In a cohort study conducted in Cape Town, postpartum HIV-positive women were offered the option of following up via a differentiated service delivery model (community adherence clubs) or at their primary care clinic [8]. The majority preferred the adherence club model, with encouraging short-term outcomes.

## Differentiated approaches to prevention

Achieving epidemic control is also critically dependent on HIV prevention. Primary prevention of HIV acquisition is required in addition to optimizing the potential of HIV treatment as a prevention tool [1]. Between 21% and 96% of new HIV infections occur among key populations and their sexual partners [9], and the enormous structural and societal barriers described above affect access to prevention services as well as treatment. Interventions to engage key populations have been shown to alleviate some of these impediments. For example, a study conducted in Kenya showed that the use of sex worker peer educators led to an increase in safer sexual behaviors and noted that individuals who participated in more peer education sessions achieved higher levels of protection [10]. Another study, also conducted in Kenya, demonstrated the feasibility of training health workers to better understand the needs of men who have sex with men [11]. Despite these successes, novel and effective strategies remain urgently needed to decrease HIV incidence amongst key and priority populations, and engaging members of these communities in designing and testing primary prevention initiatives is a priority.

## The way forward

**Fig 1:**
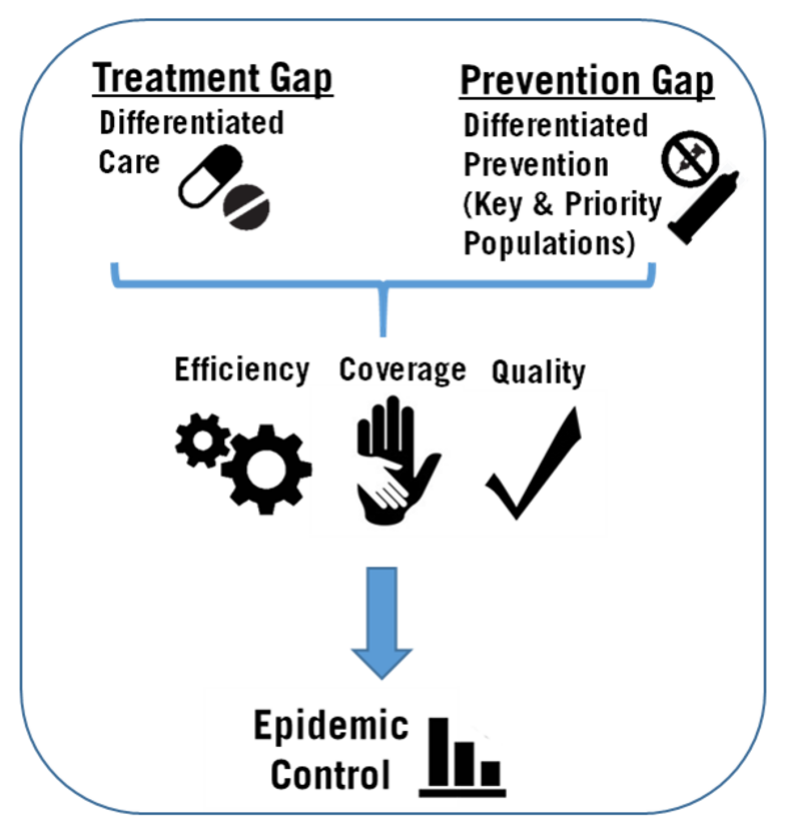
Framework for achievement of HIV epidemic control.

As the expression goes, “What got us here won’t get us there.” Attaining epidemic control will require continued rapid expansion of the number of PLHIV on treatment, engaging populations at risk for HIV infection, improvement of the quality of HIV services, and new approaches to program design and implementation (Fig 1). The scale-up of differentiated care has the potential to relieve crowded health facilities and overworked providers by moving stable patients on ART to more patient-centered models, enhancing both efficiency and quality. Differentiated care can also facilitate the engagement of other groups of PLHIV in models of service provision that meet their specific clinical and psychosocial needs. At the same time, innovations are urgently needed in the development of differentiated prevention delivery models that address the needs of specific groups at substantial risk for HIV infection. In addition, it is now more important than ever to utilize population-based, programmatic, and research data in shaping programs and prioritizing populations [12]. For instance, disaggregation of seemingly favorable national population HIV data by sex and age shows important gaps in the HIV care and HIV prevention continua for men and youth living with HIV.

It is important to note that creating a multitude of service delivery models, some overly complicated, risks undermining the public health approach that has been so vital to the success of HIV programs. Abandoning the public health approach by “overdifferentiating” may be as problematic as a one-size-fits-all approach [2]. Caution is required to avoid service models that disrupt the simple, streamlined approaches to health worker training, procurement, laboratory systems, and monitoring and evaluation strategies that were so central to successful HIV program expansion. As differentiated service delivery models are taken to scale, it will be critically important to evaluate their effects on individual and programmatic outcomes, client satisfaction, health provider productivity and satisfaction, and laboratory, procurement, and monitoring systems—as well as on the affordability and cost-effectiveness of specific models of care and prevention. Fundamentally, the essence of the public health approach is that it is anchored in the realities of resource-constrained health systems. Designing, implementing, and scaling up new service models that are person centered and informed by data and evidence will enable the achievement of high coverage, quality, and efficiency—paving the way towards epidemic control.

## Abbreviations

ART: antiretroviral therapy
PEPFAR: President’s Emergency Plan for AIDS Relief
PLHIV: persons living with HIV
